# Multimodality imaging: Bird’s eye view from The European Society of Cardiology Congress 2015 London, August 29-September 2, 2015

**DOI:** 10.1007/s12350-015-0322-1

**Published:** 2015-11-11

**Authors:** Jeroen J. Bax, Victoria Delgado, Stephan Achenbach, Udo Sechtem, Juhani Knuuti

**Affiliations:** Department of Cardiology, Heart Lung Centrum, Leiden University Medical Center, Albinusdreef 2, 2300 RC Leiden, The Netherlands; Department of Cardiology, University Hospital Erlangen, Medizinische Klinik 2, Erlangen, Germany; Department of Cardiology, Robert-Bosch-Krankenhaus Stuttgart, Stuttgart, Germany; Turku PET Centre, Turku University Hospital, University of Turku, Turku, Finland

This bird’s eye view from the 2015 European Society of Cardiology (ESC) congress hosted in London from August 29 to September 2 includes highlighted abstracts on multimodality imaging. At this ESC meeting, 407 imaging abstracts (40% of abstracts submitted under the topic of imaging) were presented. Four experts on echocardiography (VD), cardiac computed tomography (CT) (SA), cardiac magnetic resonance (CMR) (US), and nuclear imaging (JK) summarized 4 to 7 abstracts in these areas that were of most interest to them. These abstracts were integrated by one of the Editors of the Journal (JB).

## Echocardiography

Speckle tracking echocardiography (STE) continues providing novel insights into cardiac mechanics and prognosis. The Berliner Frauen Risiko evaluation study assessed the association between left atrial (LA) reservoir, conduit and booster pump functions measured with STE and left ventricular (LV) diastolic dysfunction grade in 449 women.^1^ Strain-derived reservoir and conduit functions reduced in parallel to worsening diastolic dysfunction, whereas LA booster pump function increased in grade 1 diastolic dysfunction, and subsequently decreased in grade 2 and 3 diastolic dysfunction. Compared with LA volume index, strain-derived reservoir and conduit functions showed higher sensitivity (73.7% and 87.2%, respectively) and specificity (80.4% and 75.8%, respectively) to predict LV diastolic dysfunction. These results suggest that changes in LA functions assessed with STE may precede LA remodeling in the early phases of LV diastolic function.

Early diagnosis of arrhythmogenic right ventricular dysplasia (ARVD) using STE was the objective of the study presented by Teske and coworkers.^2^ The electro-mechanical interval, defined as the time difference between first ECG-detected deflection of the QRS complex and the onset of shortening of the basal, mid, and apical segments of the right ventricular (RV) free wall, was measured as a surrogate of activation delay in 44 mutation-positive ARVD patients, 31 mutation carriers without phenotype of ARVD, and 30 healthy controls. The electro-mechanical interval was associated with the occurrence of ventricular tachyarrhythmias. From the healthy controls, a cut-off value >100 ms was derived to define an abnormal electro-mechanical interval. Mutation-positive ARVD patients had an abnormal electro-mechanical interval in all RV segments, whereas only 55% of mutation carriers without ARVD phenotype showed an abnormal electro-mechanical interval in the basal RV segment. After a median follow-up of 3.8 ± 2.8 years, patients of the mutation-positive and the mutation carriers without phenotype of ARVD groups with an abnormal electro-mechanical interval at the basal segment of the RV free wall showed a significantly higher burden of ventricular arrhythmias.

The results of a sub-study from the EchoCRT trial (Cardiac resynchronization therapy in heart failure with narrow QRS complex) were presented, focusing on the prognostic implications of persistent or worsened LV dyssynchrony after CRT implantation.^3^ Of 614 patients with echocardiographic follow-up at 6 months after CRT implantation, 77% showed persistent or worsened LV dyssynchrony (≥130 ms as measured with STE or ≥80 ms using tissue Doppler imaging). Patients with persistent or worsened LV dyssynchrony showed a significantly increased risk of all-cause mortality and heart failure hospitalization (hazard ratio 1.54, 95% confidence interval 1.03-2.30; *P* = .02) compared with patients with reduced LV dyssynchrony, confirming observational results in heart failure patients with wide QRS complex.^4^

**Figure 1:**
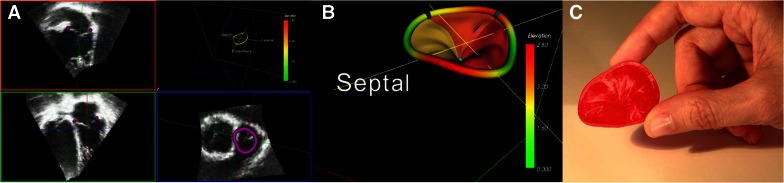
Three-dimensional printing of the tricuspid valve. From three-dimensional transthoracic echocardiographic data of the tricuspid valve, coordinates of the leaflets and annulus are demarcated (**A**) and imported to dedicated software to create the model (**B**) which is converted to stereolithographic file format and printed in 3D (**C**). Reproduced with permission from Muraru et al^6^

Three-dimensional transthoracic echocardiography (3D TTE) attracts significant attention. Tricuspid valve regurgitation in patients with RV pacing leads has poor prognosis.^5^ In 145 patients with pacemaker or implantable cardioverter defibrillator (ICD), the mobility of the RV pacing lead and its position relative to the tricuspid valve annulus was assessed with 3D TTE. At follow-up after device implantation, 30% presented with grade 3 or more tricuspid regurgitation. Patients were classified as having low risk for significant tricuspid regurgitation if the lead was freely mobile without interfering with the leaflet movement and positioned in the center of the tricuspid valve annulus or in the commissure of the tricuspid leaflets. In contrast, patients with high risk of developing significant tricuspid regurgitation had more frequently fixed leads interfering with leaflet mobility and more frequently showed leaflet perforation. Three-dimensional analysis of the tricuspid valve and the underlying pathophysiology of tricuspid regurgitation may help to personalize treatment of patients with symptomatic severe tricuspid regurgitation. The use of dedicated software to create a 3D model that can subsequently be printed may help to decide on the repair approach. Muraru and coworkers presented the first 3D printed model of the tricuspid valve.^6^ Coordinates of the tricuspid annulus and leaflets were imported into Mesh-Lab (Visual Computing Lab ISTI-CNR) software to build a model which was converted to stereolithographic file format and printed in 3D in 30 minutes (Figure 1). The 3D printed tricuspid valve permitted better perception of the complex 3D geometry.

## Computed Tomography

In cardiac CT, most of the presented research at the ESC Congress 2015 was in relation to clinical applications, and relatively few abstracts concerned new technical developments. The use of “virtual” fractional flow reserve, derived from the anatomic information provided by coronary CT angiography (CTA), received most attention. The PLATFORM study^7^ was designed to evaluate the utility of coronary CTA with fractional flow reserve (FFR-CT) to serve as a “gatekeeper” for invasive angiography. A total of 584 patients with new onset chest pain were recruited in 11 centers in Europe, with core laboratory evaluation in the United States. The patients were divided in 2 cohorts: a “non-invasive workup first” cohort of 204 individuals, and a “planned invasive angiography” cohort of 380 patients, depending on the management planned by the treating physician. In both cohorts, patients were randomized to either the originally planned test, or to coronary CTA with FFR-CT (in the presence of 30% or more luminal stenosis). The primary outcome measure was the rate of invasive coronary angiograms that did not show obstructive disease in the cohort that had been planned to undergo invasive angiography. Indeed, the use of coronary CTA (plus FFR-CT in 117 cases) resulted in a rate of invasive coronary angiograms showing no stenosis of 12.4% (24/193), as compared to 73.3% (137/187) in those who underwent the initially planned invasive angiogram directly, without prior CT. In the cohort of 204 patients for whom a non-invasive workup was initially planned, there was no significant difference in the rate of invasive angiograms showing no stenoses (6.0% without CTA/FFR-CT vs. 12.5% with CTA/FFR-CT, *P* = .95). However, this was not a primary outcome measure. Average radiation exposure was not increased by the use of CTA/FFR-CT in either cohort. Ninety-day major adverse cardiac event (MACE) rates were not increased in those patients who underwent CTA rather than invasive angiography. All in all, the study reassuringly showed that CTA/FFR-CT can reduce the number of unnecessary invasive angiograms, and specifically reduce the number of angiograms showing no stenosis, in patients with suspected coronary disease—hence well serving its “gatekeeper” role.

**Figure 2:**
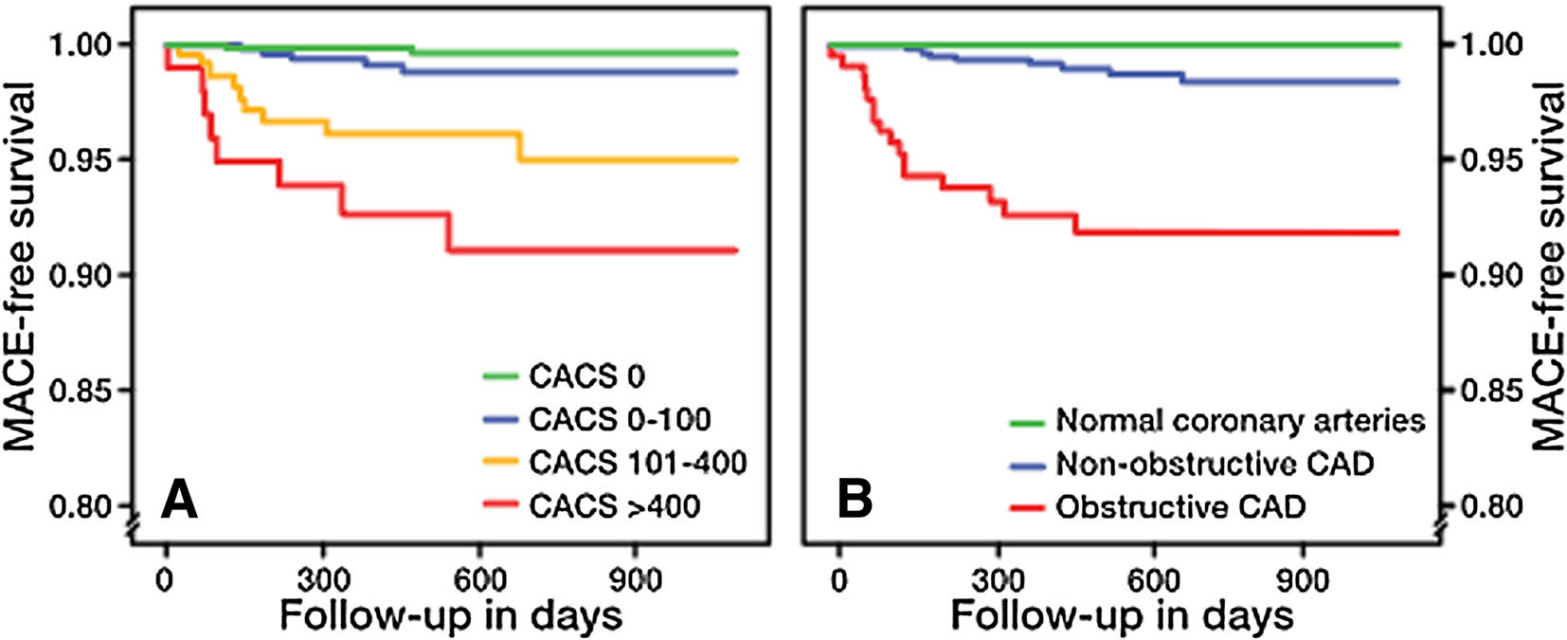
Major adverse cardiac event (MACE)-free survival in a cohort of 1551 patients with chest pain and a low-to-intermediate pre-test probability for coronary artery disease followed for a median of 637 days. A total of 23 MACE occurred, in the majority late revascularizations (*n* = 16). *Left* prognostic value of the calcium score, *right* prognostic value of coronary computed tomography angiography (CTA). In multivariable analysis, obstructive plaque in coronary CTA added significantly to the calcium score and risk factors. *CACS*, coronary artery calcium score; *CCTA*, coronary computed tomography angiography. Reproduced with permission from Bom et al^8^

Numerous abstracts dealt with the prognostic value of coronary CTA. Bom et al evaluated 1551 patients with low-to-intermediate risk of coronary artery disease who underwent coronary CTA as well as calcium scoring.^8^ During a median follow-up of 637 days, 23 patients (1.5%) experienced an event: late revascularizations (16/23), myocardial infarction (4/23), and death (3/23). Both coronary calcium scoring and coronary CTA had prognostic value. The multivariable analysis, adjusting for risk factors and coronary calcium score, demonstrated an independent prognostic value of coronary CTA (Figure 2).

Marwan et al reported the results of the German cardiac CT registry regarding the presence of coronary artery lesions in coronary CTA of 2016 individuals without coronary calcium.^9^ In 18% of these individuals, non-obstructive coronary lesions and in 2.3%, obstructive lesions were found, emphasizing that coronary atherosclerosis can occur even in patients without coronary calcium.

An increasingly important area is the use of cardiac CT for non-coronary interventions, for example, in heart valve disease. Fonseca et al presented a series of 131 patients who underwent transcatheter aortic valve implantation with a balloon-expandable (36%) or self-expanding (64%) prosthesis.^10^ 53% of patients had paravalvular regurgitation after the procedure, which was mild in 46% and moderate in 8%. Aortic valve calcium was predictive of post-implant regurgitation, with an optimal cut-off value of 157 mm^3^ of calcium measured with a lower threshold of 850 HU to accept a structure as “calcium.” The degree of aortic valve calcification (optimum cut-off: 267 mm^3^) was also a predictor for the need to perform balloon post-dilatation. Mitral valve interventions may also profit from CT imaging. In 25 patients planned for edge-to-edge mitral valve repair, CTA and transesophageal echocardiography showed excellent agreement regarding parameters such as leaflet length, annulus diameters, and left ventricular dimensions.^11^ Finally, CT imaging is important in patients considered for transcatheter LA appendage occlusion, and CT may be the preferred method for assessment of mean diameter of the LA appendage orifice.^12^

## Cardiac Magnetic Resonance Imaging

Important topics in research with cardiac magnetic resonance (CMR) at the ESC Congress 2015 included assessment of diffuse myocardial fibrosis, advanced diagnosis of coronary artery disease, and risk stratification for ICD implantation.

Validation of pre-contrast T1 mapping values and myocardial extracellular volume measurement with CMR techniques as surrogates of interstitial diffuse fibrosis was the objective of several studies including patients with different cardiomyopathies.^13^-^15^ In patients with dilated cardiomyopathy, longer pre-contrast T1 mapping values were significantly correlated with larger amounts of extracellular volume and larger histological collagen volume fraction, indicating more extensive LV fibrosis.^14^,^15^ In addition, in 531 consecutive patients referred for CMR for other reasons than hypertrophic cardiomyopathy, Kammerlander et al showed that increasing myocardial extracellular volume was independently associated with increased risk of heart failure hospitalization and cardiovascular death.^13^ In patients with acute ST-segment elevation myocardial infarction, magnitude and changes in myocardial extracellular volume (assessed with T1 mapping) in areas remote from the infarct core, were associated with changes in LV ejection fraction (EF).^16^ In 171 ST-segment elevation myocardial infarction patients, extracellular volume of the remote area at baseline (2 days after index infarction) and at 6 months follow-up did not change significantly (from 25.6 ± 3.1% to 25.5 ± 2.8%), whereas LVEF increased from 55.6 ± 9.3% to 62.7 ± 9.4%. A change in extracellular volume was inversely associated with a change in LVEF (*r* = −0.23; *P* = .011). Moreover, an increase in extracellular volume of the remote area of ≥1% at 6 months follow-up was observed in 43% of patients.^17^ Compared with patients in whom extracellular volume did not change, patients with increased extracellular volume showed “higher risk” characteristics: older age, higher peak troponin, more frequent microvascular obstruction and reduced LVEF, and larger LV volumes at follow-up. This work demonstrates that the adverse process of LV remodeling post-ST-segment elevation myocardial infarction can be detected and quantified non-invasively with CMR.

**Figure 3:**
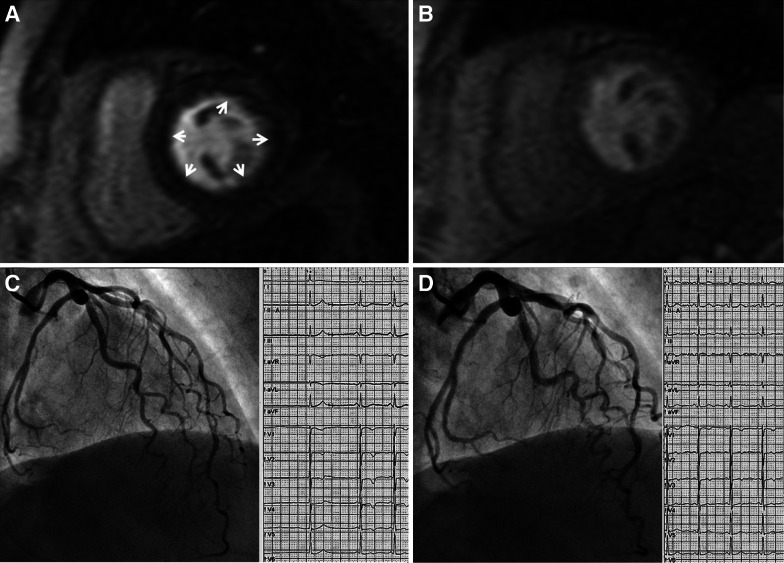
Stress CMR to assess microvascular disease. Example of a 72-year-old woman with angina complaints during rest and negative troponin. After reaching a peak exercise of 75 W during electrocardiogram stress testing, the patient developed chest pain. There was a ST-segment depression in I, II, aVF, V4 to V6 leads. CMR showed circular subendocardial hypoenhancement during adenosine stress (**A**, *arrows*). Perfusion at rest was normal (**B**). Acetylcholine testing at 100 µg showed diffuse mild constriction of the epicardial left coronary artery (**C**) which was accompanied by ST-segment depression in V1 to V3 leads. Following nitroglycerin injection into the left coronary artery, the epicardial coronary artery appeared wider (**D**) and ST-segment abnormalities resolved. Reproduced with permission from Ong et al^19^

Coronary microvascular disease is characterized by angina, positive tests for ischemia, and the absence of epicardial coronary stenoses. CMR can be useful in these patients to determine coronary flow reserve by measuring coronary sinus flow at rest and during pharmacologic stress, and then myocardial perfusion reserve is calculated as the ratio between stress coronary sinus flow/baseline coronary sinus flow in 245 consecutive patients. Patients with a myocardial perfusion reserve below or equal to the mean (2.75) had worse outcome compared to patients with preserved myocardial perfusion reserve (>2.75).^18^ Furthermore, patients with microvascular dysfunction often experience resting chest pain due to concomitant microvascular spasm. Coronary spasm including microvascular spasm can be provoked by intracoronary acetylcholine. This test revealed microvascular spasm in 59 out of 125 patients, whereas epicardial spasm was elicited in 28 of 125 patients.^19^ Stress CMR showed a reversible adenosine-induced subendocardial perfusion defect in 58 out of these 125 patients (Figure 3).

Patients with dilated cardiomyopathy and LVEF<35% are candidates for ICD implantation as primary prevention of sudden cardiac death. The incremental prognostic value of LVEF and the presence of myocardial scar assessed with CMR were evaluated in 270 patients with dilated cardiomyopathy.^20^ During 2.5 years of follow-up, 68 presented with ventricular tachyarrhythmias and sudden cardiac death. Patients with events had lower LVEF (29 ± 10 vs. 32 ± 9%, *P* = .003) and higher frequency of myocardial scar (67 vs. 44%, *P* = .0009) than patients who did not present with events.

In addition, an interesting multicenter trial studied whether the extent and heterogeneity of late gadolinium enhancement (LGE) areas (identifying the infarct border zone) predicted appropriate ICD therapy in 55 patients with ischemic cardiomyopathy and 62 with dilated cardiomyopathy.^21^ During a mean follow-up of 4 years, patients with larger (above the median) compared to smaller border zones had higher rates of ICD therapy, whereas total LGE burden was similar between groups with and without ICD therapy. The importance of the size of the border zone surrounding the scar was confirmed in another study of 138 ST-segment elevation myocardial infarction patients who were studied by CMR within 1 week post-infarction.^22^ In a multivariate model that included clinical and other established prognostic parameters, the extent of the border zone was an independent predictor of the combined clinical endpoint (all-cause death, re-infarction, new congestive heart failure within 1 year of infarction). Thus, border zone size may be a marker for risk stratifying patients eligible for primary prevention ICD therapy.

## Nuclear Cardiology

In nuclear cardiology, not only a large fraction of abstracts focused on coronary artery disease and myocardial perfusion imaging (MPI) but also other methods such as innervation imaging gained increasing attention.

Al-Mallah et al^23^ investigated the incremental prognostic significance of coronary flow reserve in 2645 consecutive patients with known or suspected coronary artery disease using positron emission tomography (PET, stress, and rest) and rubidium-82. During a median follow-up of 1.4 years, data on cardiac death or myocardial infarction were collected. The lowest tertile of coronary flow reserve (<1.8) was associated with a 3.7-fold increase in the risk of events, as compared with the highest tertile. This remained significant after adjusting for clinical variables, perfusion defect size, and resting LVEF. The study further emphasizes the clinical value of absolute quantification of myocardial perfusion and perfusion reserve over standard visual assessment.

The prognostic value of single-photon emission computed tomography (SPECT) MPI was addressed in 564 patients who were evaluated for renal transplantation.^24^ During a median follow-up of 43.5 months, 122 patients died (9.6% of cardiovascular causes). Compared with patients without ischemia, patients with mild (5-10% of the LV myocardium) and with substantial ischemia (>10%) showed a significantly increased mortality risk (respective hazard ratios: 2.27 and 2.44). However, patients with substantial ischemia had lower risk for cardiovascular death than patients with mild ischemia, possibly because of more frequent revascularizations.

The detection of vulnerable plaques using non-invasive imaging has gained a lot of interest over the recent years. PET imaging using F-18-fluorodeoxyglucose (FDG) has been used in numerous studies to detect vascular inflammation in the aorta and carotid arteries. Recently, studies have used FDG and F-18-fluoride to detect active plaques in human coronary arteries. Locorotondo et al^25^ compared the characteristics of unstable coronary artery plaques detected by intracoronary optical coherence tomography and FDG PET imaging in 20 consecutive patients with single-vessel coronary artery disease. Coronary FDG uptake was enhanced in patients with non-ST-elevation acute coronary syndrome as compared to patients with stable coronary artery disease, with a trend toward higher activity in patients with inflamed thin cap fibro-atheromas. The majority of patients (88%) without inflamed thin cap fibro-atheromas and low FDG uptake had stable coronary artery disease, while all patients with inflamed thin cap fibro-atheromas and high FDG uptake had non-ST-elevation acute coronary syndrome suggesting that FDG uptake in plaques is associated with inflammation and vulnerability.

**Figure 4:**
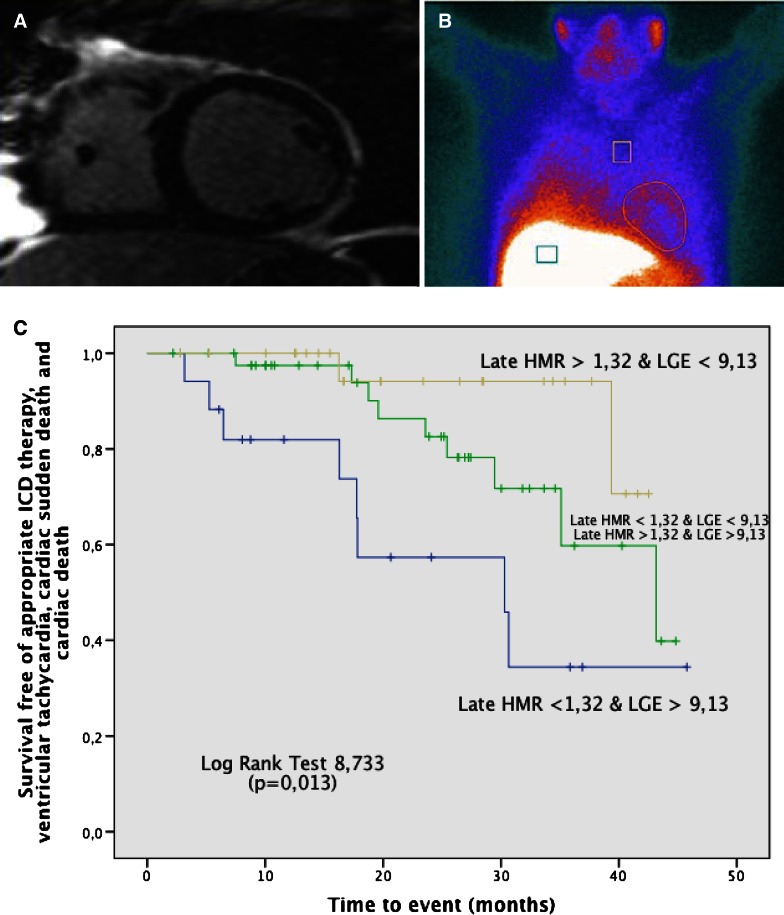
Combination of fibrosis imaging by cardiac magnetic resonance (CMR) (**A**) and late and I-123-iodine metaiodobenzylguanidine (MIBG) uptake (**B**) and outcomes (**C**): patients with larger amount of late gadolinium enhancement (LGE) on CMR and lower MIBG uptake on SPECT have worse prognosis than the other groups of patients. Reproduced with permission from García-González et al^26^

Several studies focused on cardiac innervation imaging using SPECT and I-123-iodine metaiodobenzylguanidine (MIBG) or PET with [11C]hydroxyephedrine (HED). Garcia Gonzalez et al^26^ performed scar imaging with CMR and planar MIBG imaging in 86 heart failure patients who were referred for ICD implantation. The endpoints included appropriate ICD shocks, ventricular tachycardia, and cardiac death; 19 patients (22%) reached the endpoint. These patients had larger scar areas on late gadolinium enhancement (LGE ≥9.13%) and reduced MIBG uptake (heart to mediastinum ratio ≤1.32). The combination of LGE CMR and MIBG imaging improved the risk stratification (Figure 4).

Exercise training has beneficial effects in heart failure patients. Valborgland et al evaluated whether cardiac MIBG uptake would improve after 3 months of exercise training.^27^ The study enrolled 23 patients who were randomized to regular exercise, continuous training, and interval training. All patients underwent MIBG imaging before and after the training period. The authors reported no differences between the groups, suggesting that alterations in sympathetic innervation are not related to the beneficial effects of exercise training in heart failure patients.

Finally, the effect of beta-blocker therapy on cardiac sympathetic nerve function in patients with idiopathic pulmonary arterial hypertension was studied by Rijnierse et al.^28^ Patients with pulmonary arterial hypertension and right ventricular failure are known to suffer from impaired cardiac sympathetic innervation, which may be related to the severity of the disease. The authors investigated 18 patients with pulmonary hypertension using [11C]HED PET in a prospective, double-blind placebo-controlled crossover trial. A heterogeneous pattern of sympathetic nerve dysfunction was noted, particularly in the right ventricle and septum. Beta-blocker therapy resulted in increased septum and right ventricle [11C]HED uptake, suggesting a positive effect on regional neuronal remodeling.
